# Decreased Rhes mRNA levels in the brain of patients with Parkinson’s disease and MPTP-treated macaques

**DOI:** 10.1371/journal.pone.0181677

**Published:** 2017-07-25

**Authors:** Francesco Napolitano, Emily Booth Warren, Sara Migliarini, Daniela Punzo, Francesco Errico, Qin Li, Marie-Laure Thiolat, Angelo Luigi Vescovi, Paolo Calabresi, Erwan Bezard, Micaela Morelli, Christine Konradi, Massimo Pasqualetti, Alessandro Usiello

**Affiliations:** 1 Ceinge Biotecnologie Avanzate, Naples, Italy; 2 Department of Molecular Medicine and Medical Biotechnology, University of Naples Federico II, Naples, Italy; 3 Department of Pharmacology, Vanderbilt University, Nashville, TN, United States of America; 4 Department of Biology Unit of Cell and Developmental Biology, University of Pisa, Pisa, Italy; 5 Department of Environmental, Biological and Pharmaceutical Sciences and Technologies, University of Campania, Luigi Vanvitelli, Italy; 6 Motac Neuroscience, UK-M15 6WE, Manchester, United Kingdom; 7 Institute of Lab Animal Sciences, China Academy of Medical Sciences, Beijing, China; 8 Université de Bordeaux, Institut des Maladies Neurodégénératives,Bordeaux, France; 9 Centre National de la Recherche Scientifique Unité Mixte de Recherche 5293, Institut des Maladies Neurodégénératives, Bordeaux, France; 10 IRCSS Casa Sollievo della Sofferenza, ISBReMIT-Institute for Stem Cell Biology, Regenerative Medicine and Innovative Therapies, San Giovanni Rotondo, Italy; 11 Department of Medicine, University of Perugia and Clinica Neurologica, Santa Maria della Misericordia Hospital, Perugia, Italy; 12 National Research Council of Italy (CNR), Neuroscience Institute, Cagliari, Italy; 13 Department of Biomedical Sciences, section of Neuropsychopharmacology, University of Cagliari, Cagliari, Italy; 14 Center for Neuroscience and Cognitive Systems, Istituto Italiano di Tecnologia, Rovereto, TN, Italy; 15 Neuroscience Institute, National Research Council (CNR), Pisa, Italy; Hudson Institute, AUSTRALIA

## Abstract

In rodent and human brains, the small GTP-binding protein Rhes is highly expressed in virtually all dopaminoceptive striatal GABAergic medium spiny neurons, as well as in large aspiny cholinergic interneurons, where it is thought to modulate dopamine-dependent signaling. Consistent with this knowledge, and considering that dopaminergic neurotransmission is altered in neurological and psychiatric disorders, here we sought to investigate whether Rhes mRNA expression is altered in brain regions of patients with Parkinson’s disease (PD), Schizophrenia (SCZ), and Bipolar Disorder (BD), when compared to healthy controls (about 200 *post-mortem* samples). Moreover, we performed the same analysis in the putamen of non-human primate *Macaca Mulatta*, lesioned with the neurotoxin 1-methyl-4-phenyl-1,2,3,6-tetrahydropyridine (MPTP). Overall, our data indicated comparable Rhes mRNA levels in the brain of patients with SCZ and BD, and their respective healthy controls. In sharp contrast, the putamen of patients suffering from PD showed a significant 35% reduction of this transcript, compared to healthy subjects. Interestingly, in line with observations obtained in humans, we found 27% decrease in Rhes mRNA levels in the putamen of MPTP-treated primates. Based on the established inhibitory influence of Rhes on dopamine-related responses, we hypothesize that its striatal downregulation in PD patients and animal models of PD might represent an adaptive event of the dopaminergic system to functionally counteract the reduced nigrostriatal innervation.

## Introduction

Rhes (RASD2) is a gene encoding for a GTP-binding protein highly enriched in the corpus striatum [1–3], and developmentally regulated by thyroid hormones [4, 5]. To a lesser extent, also dopamine regulates Rhes mRNA levels in adult rat striatum [6, 7]. Neuroanatomical observations, performed on human and rodent brains, showed that Rhes mRNA is localized in virtually all dopamine D1 and D2 receptor (R)-bearing medium-sized spiny neurons, as well as in cholinergic interneurons of the caudate-putamen [8–10]. Rhes transcript is also expressed in rodent and human hippocampus, within the granule cell layer of the dentate gyrus and pyramidal cell layer of Ammon’s horn [1, 2, 7, 8, 11]. Interestingly, in mice Rhes mRNA-positive neurons are distributed within the cortical layers II-III, while in the human cortex Rhes transcript is detected throughout layers II–VI, with a distinctive higher expression in layer V [12]. From a functional perspective, Rhes modulates striatal dopamine D1R and D2R transmission, by influencing cAMP/PKA and AKT pathways [8–11, 13–16]. Rhes also binds to and activates mTORC1 signalling in the striatum, which is known to influence the expression of L-DOPA-induced dyskinesia (LID) in rodent PD models [17, 18]. Accordingly, we recently found that dopamine-depleted Rhes knockout mice show a significant LID reduction, while the beneficial anti-akinetic effect of L-DOPA is preserved [19, 20]. Strikingly, Snyder and colleagues reported that Rhes acts as a unique striatal E3 ligase for mutant huntingtin sumoylation, a biochemical process that causes cellular neurotoxicity in Huntington’s disease [21–26]. Thus, in addition to its involvement in PD-related processes, Rhes is currently considered to be a molecular determinant in cellular and behavioural Huntington’s disease-associated phenotypes [27]. Though a putative link between Rhes and neurodegenerative disorders has been established, this G-protein, by regulating AKT and cAMP/PKA signalling, might also play a role in biochemical pathways in the brain of BD and SCZ patients [28–30]. Accordingly, a variant of the Rhes gene (rs736212) has been associated with a sub-group of patients with SCZ [31], while another polymorphism (rs6518956) relevant for SCZ correlates with fronto-striatal endophenotypes in healthy subjects [12]. Consistent with its potential role in psychiatric diseases, Rhes also interacts with striatal proteins, such as PDE2A (phosphodiesterase 2A) and LRRC7 (leucine-rich repeat–containing 7), reported to be associated with major depression and BD [32]. Therefore, in order to investigate the possible alteration of Rhes mRNA expression in neurodegenerative and psychiatric disorders, we evaluated whether its transcript levels might be affected by the diagnosis of PD, SCZ and BD. To further dissect whether Rhes gene expression might be influenced by the dopamine denervation in an experimental non-human primate model of PD, we also evaluated striatal levels of Rhes mRNA in *Macaca Mulatta*, treated with MPTP.

## Materials and methods

All human tissue collection and processing was carried out in accordance with the declaration of Helsinki. In Italy there is no law regulating the use of biological samples obtained from internationally recognized brain banks. Therefore, because our study did not involve pharmacological research, which is regulated in Italy, ethics committee approval was not required.

Non-human primate studies were carried out in accordance with European Communities Council Directive (2010/63/EU) for care of laboratory animals in an AAALAC-accredited facility following acceptance of study design by the Institute of Lab Animal Science IACUC (Chinese Academy of Medical Sciences, Beijing, China).

### Human tissue collection

Putamen samples of normal controls and PD patients were obtained from the Harvard Brain Tissue Resource Center (HBTRC; http://www.brainbank.mclean.org/). The clinical and neuropathological criteria for a diagnosis of PD are summarized in Table 1. Dorsolateral prefrontal cortex and hippocampus samples of patients with SCZ and non-psychiatric controls were obtained from The Human Brain and Spinal Fluid Resource Center-Los Angeles (HBSFRC). Prefrontal cortex and putamen samples of patients with SCZ and non-psychiatric controls were obtained from MRC London Neurodegenerative Diseases Brain Bank (MRC-LNDBB). Anterior cingulate cortex samples of patients with SCZ, BD, and non-psychiatric controls, were donated by The Stanley Medical Research Institute (SMRI) brain collection. SCZ and BD diagnoses were established according to DSM IV criteria (see Table 2). For more information about medication of psychiatric patients see S1 and S2 Tables. All human data were anonymized.

**Table 1:** Demographic table of PD brain samples from the Harvard Tissue Resource Center.

ID	Diagnostic group	Age (years)	PMI (hours)	Accumulated lifetime L-dopa (g)	Treatment duration (y)	Average L-dopa per year treated (g)	Years diagnosed	Neurofibrillary degeneration (Braak Stage)	Cause of death
1	NC	70	14.3						myocardial infarction
2	NC	71	14.07						Unknown
3	NC	72	21.62						myocardial infarction
4	NC	72	18.25						cardiac Ischemia
5	NC	73	19.42						chronic obstructive pulmonary disease
6	NC	73	12						Cancer
7	NC	73	7.58						cardiac Arrest
8	NC	74	22.5						chronic obstructive pulmonary disease
9	NC	74	14.33						myocardial infarction
10	NC	74	18.58						myocardial infarction
11	NC	76	21.25						cardiopulmonary arrest
12	NC	77	25.23						myocardial infarction
13	NC	79	20.5						chronic obstructive pulmonary disease
14	NC	79	20.81						motor vehicle accident
15	NC	82	14.33						liver cancer
n = 15 (mean±SEM)	74.87±0.804	17.94±1.194							
16	DYS	64	11.4	1,971	11	179	11	4	cardiopulmonary arrest
17	DYS	71	5	128	1	128	8	1	cardiopulmonary arrest
18	DYS	73	18	1,074	4	234	14	2	cardiopulmonary arrest
19	DYS	75	13.3	1,752	8	219	11	1	cardiopulmonary arrest
20	DYS	77	20.7	4,663	18	267	21	3	bladder cancer
21	DYS	78	14.5	2,197	9	233	11	2	pneumonia
22	DYS	79	23.4	1,476	8	197	8	3	cardiopulmonary arrest
23	DYS	81	10.5	1,026	8	131	13	3	cardiopulmonary arrest
24	DYS	87	11.7	2,373	13	183	13	3	cardiopulmonary arrest
25	DYS	88	26.5	1,835	5	361	10	3	anoxic encephalopathy
n = 10 (mean±SEM)	77.3±2.3	15.5±2.1	1,850±375.9	8.5±1.5	213.2±21.6	12±1.2	2.5±0.3		
26	Non-DYS	71	24.7	3,011	11	274	11	1	pneumonia
27	Non-DYS	75	14.1	1,232	5	235	12	2	PD
28	Non-DYS	75	20	511	4	120	5	2	liver cancer
29	Non-DYS	75	5.5	905	6	156	6	2	bladder cancer
30	Non-DYS	75	19.8	584	4	146	7	2	pneumonia
31	Non-DYS	75	16.5	548	4	137	12	3	colon cancer
32	Non-DYS	75	17.2	219	2	110	5	2	congestive heart failure
33	Non-DYS	77	15.2	584	5	117	5	1	bradycardia, heart blockage
34	Non-DYS	77	7.2	143	1	101	5	2	bladder cancer
35	Non-DYS	79	8.2	892	6	150	17	2	cardiopulmonary arrest
36	Non-DYS	80	20.4	2,473	8	297	11	4	cardiopulmonary arrest, pneumonia
37	Non-DYS	84	19.9	24	0	146	9	4	myocardial infarction
n = 12 (mean±SEM)	76.5±0.9	15.7±1.7	927.2±265.6	4.7±0.9	165.8±19	8.7±1.1	2.2±0.3		
NC vs PD (DYS+Non-DYS) (*p value*)	0.0672	0.2494							
DYS vs Non- DYS (*p value*)	0.6566	0.8718	0.0408	0.0391	0.1187	0.0642	0.4063		

Abbreviations: NC, non-PD control; DYS, dyskinetic PD; Non-DYS, non-dyskinetic PD; PMI, post-mortem interval; Mann-Whitney U test for DYS vs non-DYS characteristics

**Table 2:** Demographic table of psychiatric brain samples.

**The Human Brain and Spinal Fluid Resource Center (Los Angeles, CA)**						
	**NC group**	**SCZ group**				p value
	n = 20 (median; sum of ranks)	n = 20 (median; sum of ranks)				
Age	73.5; 555	52.5; 265				< 0.0001
PMI (h)	12.85; 335	15.25; 485				0.0422
pH	6.545; 223.5	6.5; 182.5				0.3574
Gender	16 M, 4 F	12M, 8 F		χ^2^ = 1.905		0.1675
Diagnosis		DSMIV-TR				
**MRC London Neurodegenerative Diseases Brain Bank**						
	**NC group**	**SCZ group**				p value
	n = 12 (median; sum of ranks)	n = 10 (median; sum of ranks)				
Age	63.5;140.5	63; 112.5				0.8836
PMI (h)	31.5; 95	48; 136				0.0070
Gender	6M, 6F	6M, 4F		χ^2^ = 0.2200		0.6390
Diagnosis		DSMIV-TR				
**The Stanley Medical Research Institute**						
	**NC group**	**SCZ group**	**BD group**			p value
	n = 32 (median;sum of ranks)	n = 36 (median;sum of ranks)	n = 29 (median;sum of raks)			
Age	44.5; 1109	43.5; 12.38			NC vs SCZ	0.9586
	44.5; 963		44; 928		NC vs BD	0.6799
PMI (h)	28; 1059	30; 1288			NC vs SCZ	0.5803
	28; 862.5		35; 1029		NC vs BD	0.0614
Gender	23M, 9F	27M, 9F	14M, 15F	χ^2^	0.0850	0.7706
				(NC vs SCZ)		
				χ^2^	3.550	09.55.00
				(NC vs BD)		
Diagnosis		DSMIV-TR				

Abbreviations: NC, non-psychiatric control; SCZ, schizophrenia; BD, bipolar disorder; PMI, postmortem interval; h, hours; M/F, male/female; Mann-Whitney U test for Age and PMI characteristics. Chi-square test for Gender characteristic. DMS, Diagnostic and Statistical Manual of mental disorders

### Non-human primates housing

Captive bred female macaques (*Macaca mulatta*, Xierxin, Beijing, PR of China; mean age = 5 ± 1 years; mean weight = 5.3 ± 0.8 kg), were housed in individual primate cages under controlled conditions of humidity (50 ± 5%), temperature (24 ± 1°C), and light (12 h light/12 h dark cycles, time lights on 8:00 am), and allowing visual contacts and interaction with macaques housed in the adjacent cages. Food and water were available ad libitum and animal care was supervised daily by veterinarians skilled in the healthcare and maintenance of non-human primates.

### MPTP-treated non-human primate PD model

Out of fifteen macaques, five received daily MPTP hydrochloride injections (0.2mg/kg, intravenously) until parkinsonian signs appeared [33]. Once PD motor signs were stable, five more animals were treated twice daily with an individually titrated dose of L-DOPA that provided maximum reversal of parkinsonian motor signs (Madopar, L-DOPA/carbidopa, 4:1 ratio; range, 9–17mg/kg). This dose of L-DOPA, defined as 100% dose, was used for chronic L-DOPA treatment, which lasted 4 to 5 months until dyskinesia stabilized. Animals then received L-DOPA twice a week to maintain a consistent level of dyskinesia before acute drug tests were carried out using a within-subject experimental design. The remaining five monkeys were used as control. Killing of animals and processing of tissues were carried out according to a previous protocol [33]. Briefly, all the animals were killed by sodium pentobarbital overdose (150 mg/kg, i.v.), and the brains were quickly removed after death. Each brain was bisected along the midline, and the two hemispheres were immediately frozen by immersion in isopentane (-45°C) and then stored at -80°C. Tissue was sectioned coronally at 20 μm in a cryostat at -17°C, thaw-mounted onto gelatin-subbed slides, dried on a slide warmer, and stored at -80°C.

### *In situ* hybridization (ISH)

ISH analysis was performed on coronal sections from adult macaque brains (n = 3 control group), according to a protocol previously described [12]. RASD2 cDNA was cloned by PCR using the following primers: forward 5’-TACCAGCTGGACATCCTGG-3’, reverse 5’-CGTCACCGTACTGCACGG-3’. RASD2 35S-labelled antisense riboprobe, 433 bp in length (nucleotides 398–830, XM-015150119), was used ISH analysis. Hybridized sections were exposed to Biomax MR X-ray films (Kodak, Rochester, NY) for three days. Images of autoradiography films of radioactive ISH experiments were scanned at a resolution of 4800 dpi and images at high magnification were acquired using bright field light microscopy [34].

### Quantitative RT-PCR analysis in human *post mortem* brain samples and macaques

Total RNAs from the HBSFRC, MRC-LNDBB, and SMRI brain samples were extracted using RNeasy^®^ Mini kit (Quiagen, Hilden, Germany), according to the manufacturer’s instructions. We used 0.5 μg of total RNA per sample to synthesize cDNA. Quantitative RT-PCR with Real Time ready catalog Assays (hRhes forward: GGGAGCCACCACAGACTC and hRhes reverse: CTGGACAAAGTCTTCATCATGG; GAPDH forward: AGCCACATCGCTCAGACAC and GAPDH reverse: GCCCAATACGACCAAATCC; ACTB forward: TCCTCCCTGGAGAAGAGCTA and ACTB reverse: CGTGGATGCCACAGGACT; PPIA forward: TTCATCTGCACTGCCAAGAC and PPIA reverse: CACTTTGCCAAACACCACAT) and LightCycler^®^ 480 Probe Master (Roche Diagnostics, Indianapolis, IN) was performed on a Light Cycler 480 II Real Time PCR system with 96-well format (Roche Diagnostics). All measurements from each subject were performed in duplicate. Rhes transcript quantities were normalized by the geometric mean of three housekeeping genes for analysing samples from MRC-LNDBB and SMRI (ACTB, GAPDH and PPIA), and two for the samples of the HBSFRC (ACTB and PPIA). After total RNA extraction (0.5 μg) from macaque tissue, quantitative Real Time amplifications were performed with LightCycler 480 SYBR Green I Master (Roche Diagnostic), in a LightCycler 480 Real Time thermocycler (Roche). The following protocol was used: 10 s for initial denaturation at 95°C followed by 45 cycles consisting of 10 s at 94°C for denaturation, 10 s at 65°C for annealing, and 6 s for elongation at 72°C temperature. The following primers were used for Rhes cDNA amplification: MmRhes forward: TAACCGGGAGTCCTTCGATG and MmRhes reverse: CCTCAAAGTAGGCGCAGTTC; Mmb-Actb forward: CTGTGCTATGTCGCCCTAGA and Mmb-Actb reverse: GGAAGGTTGGAAGAGAGCCT. Rhes transcript quantities were normalized by the geometric mean of three housekeeping genes [12, 35, 36]: MmPp1a forward: TGCTGGACCCAACACAAATG and MmPp1a reverse: GTCCACAGTCAGCAATGGTG; MmGAPDH forward: AGGTCGGAGTCAACGGATTT and MmGAPDH reverse: ATCTCGCTCCTGGAAGATGG. Total RNA (150 ng) was extracted from the Putamen of HBTRC samples using the RNeasy Mini Kit (Qiagen). DNA was eliminated from the sample by treatment with DNase I (New England Biolabs, Ipswich, MA). cDNA was synthesized from 150 ng RNA with the Superscript IV kit and random hexamers (Thermo Fisher Scientific, Waltham, MA). Quantitative Real Time PCR amplifications were performed with KAPA SYBR Fast, with ROX as a loading control (Roche Diagnostics), in the MX3005P thermocycler (Agilent Technologies, Wilmington, DE), under the following cycle conditions: 10 min at 95°C for initial denaturation followed by 40 cycles of 20 s at 95°C for denaturation, 20 s at 60°C for annealing, and 20 s at 74°C for elongation. The following primers were used for Rhes cDNA amplification: hRHES-F: TCCATCGTGTCTCGCTTCCT and hRHES-R: CTCATCGAAGGACTCCCGGT. Rhes transcript quantities were normalized by the geometric mean of five housekeeping genes [12, 35, 36]: Human 18s rRNA-F: ACGGCCGGGGGCATTCGTAT, Human 18s rRNA-R: ATCGCCGGTCGGCATCGTTT; hITM2B-F: CATTGTTATGCCACCCAGAA, hITM2B-R: GCAGTTTGTAAGTTTCCTTGTCA; hCANX-F: ACTTGTGTTGATGTCTCGGGC, hCANX-R: CGTTTTGGGGTTTTTGTGTCGG; hActb-F: AGAGCCTCGCCTTTGCCGATCC, hActb-R: CATGCCGGAGCCGTTGTCGAC; hEIF1-F: GTGATGACCTGCTTCCTGCT, hEIF1-R: CTTGCGTTGGTCACCCTGTA.

Data were analysed through Kruskal-Wallis test and/or Mann-Whitney U test, by using GraphPad software (La Jolla, CA).

## Results

### Expression levels of Rhes mRNA in Parkinson’s disease, Schizophrenia and Bipolar Disorder human brains

**Fig 1:**
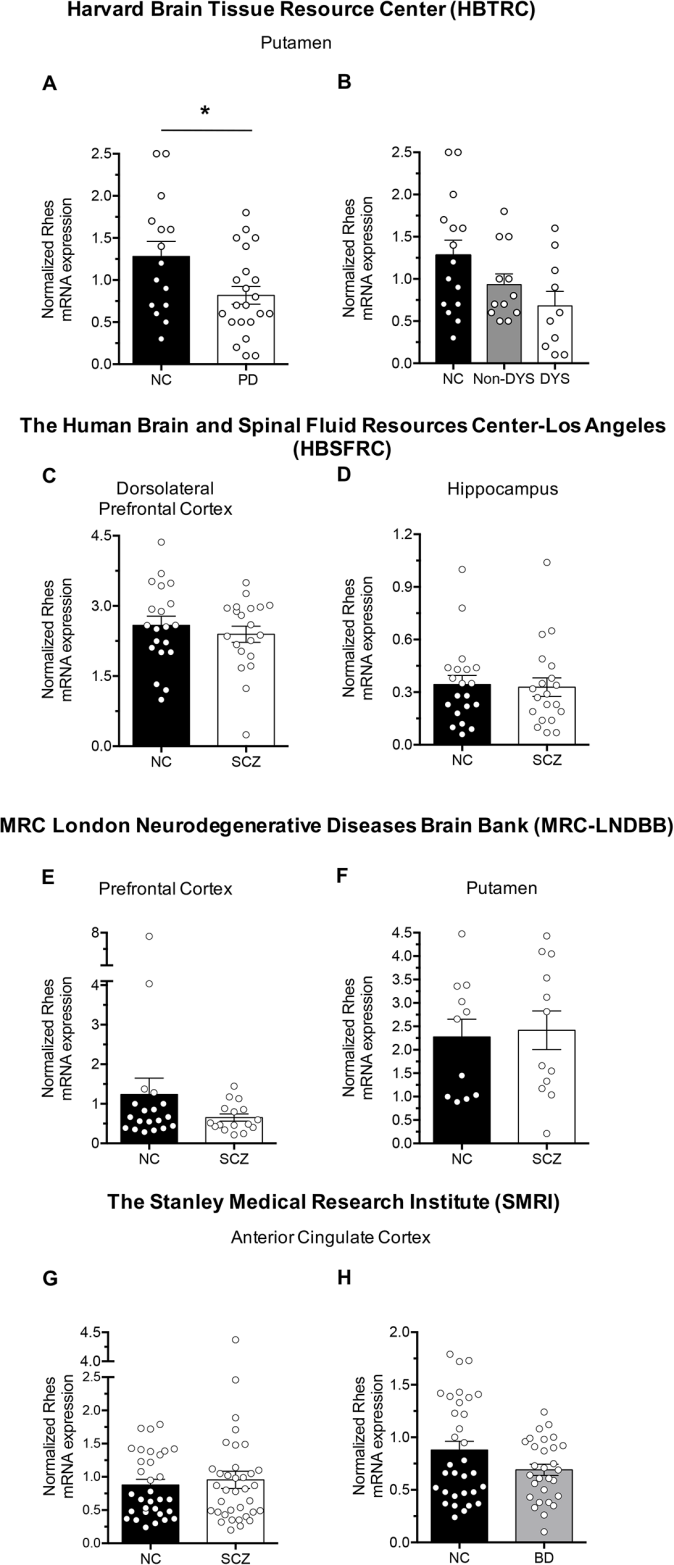
Quantitative RT-PCR analysis shows a selective reduction in Rhes mRNA levels in *post- mortem* brain samples of patients with Parkinson’s disease. (A) Putamen samples of PD patients (n = 22), and non-PD controls (n = 15), from the HBTRC. (B) Putamen samples of non-dyskinetic (n = 12) and dyskinetic (n = 10) patients and non-PD controls, from the HBTRC. (C, D) Dorsolateral prefrontal cortex (C) and hippocampus (D) samples from patients with SCZ (n = 20) and non-psychiatric controls (n = 20), from the HBSFRC. (E) Prefrontal cortex samples of patients with SCZ (n = 16) and non-psychiatric controls (n = 19), from MRC-LNDBB. (F) Putamen samples of patients with SCZ (n = 12) and non-psychiatric controls (n = 11), from MRC-LNDBB. (G, H) Anterior cingulate cortex samples of patients with SCZ (n = 36) (G), BD (n = 29) (H) and non-psychiatric controls (n = 32), from the SMRI. Data were analysed by using Kruskal-Wallis test or Mann-Whitney U test. * *p*<0.05, compared to non-pathological control (NC) group. All values are expressed as mean ± SEM.

We analysed Rhes mRNA levels, by qPCR analysis, in different cerebral regions of patients affected by PD, SCZ and BD (See Tables 1 and 2 for demographic details), obtained from four different human brain banks. Notably, our data showed a significant decrease in Rhes mRNA amount in the putamen of PD patients from the HBTRC, compared to the control group (*P* = 0.0420; Mann-Whitney U test; Fig 1A). Since PD group consists of dyskinetic and non-dyskinetic patients, we also compared Rhes mRNA levels among these groups. Overall, Kruskal-Wallis analysis revealed a clear trend toward reduction of Rhes expression in both dyskinetic and non-dyskinetic patients, compared to controls (*P* = 0.0634; Fig 1B).

Conversely, comparable levels of Rhes mRNA were found between SCZ patients and healthy controls, in both dorsolateral prefrontal cortex (*P* = 0.6783; Mann-Whitney U test; Fig 1C) and hippocampus (*P* = 0.7632; Fig 1D) samples from the HBSFRC. Similar results were also obtained in both prefrontal cortex (*P* = 0.4611; Mann-Whitney U test; Fig 1E) and putamen of SCZ patients (*P* = 0.5254; Fig 1F) from the MRC-LNDBB. In addition, qPCR analysis in the anterior cingulate cortex of SCZ brains from the SMRI, displayed no difference in Rhes mRNA levels, when compared to non-psychiatric controls (*P* = 0.9246; Mann-Whitney U test; Fig 1G). Finally, analysis of mRNA levels in the same brain region of patients with BD, obtained from the SMRI, revealed no difference when compared to non-psychiatric controls (*P* = 0.2254; Fig 1H).

### Localization of Rhes mRNA in macaque brain

**Fig 2:**
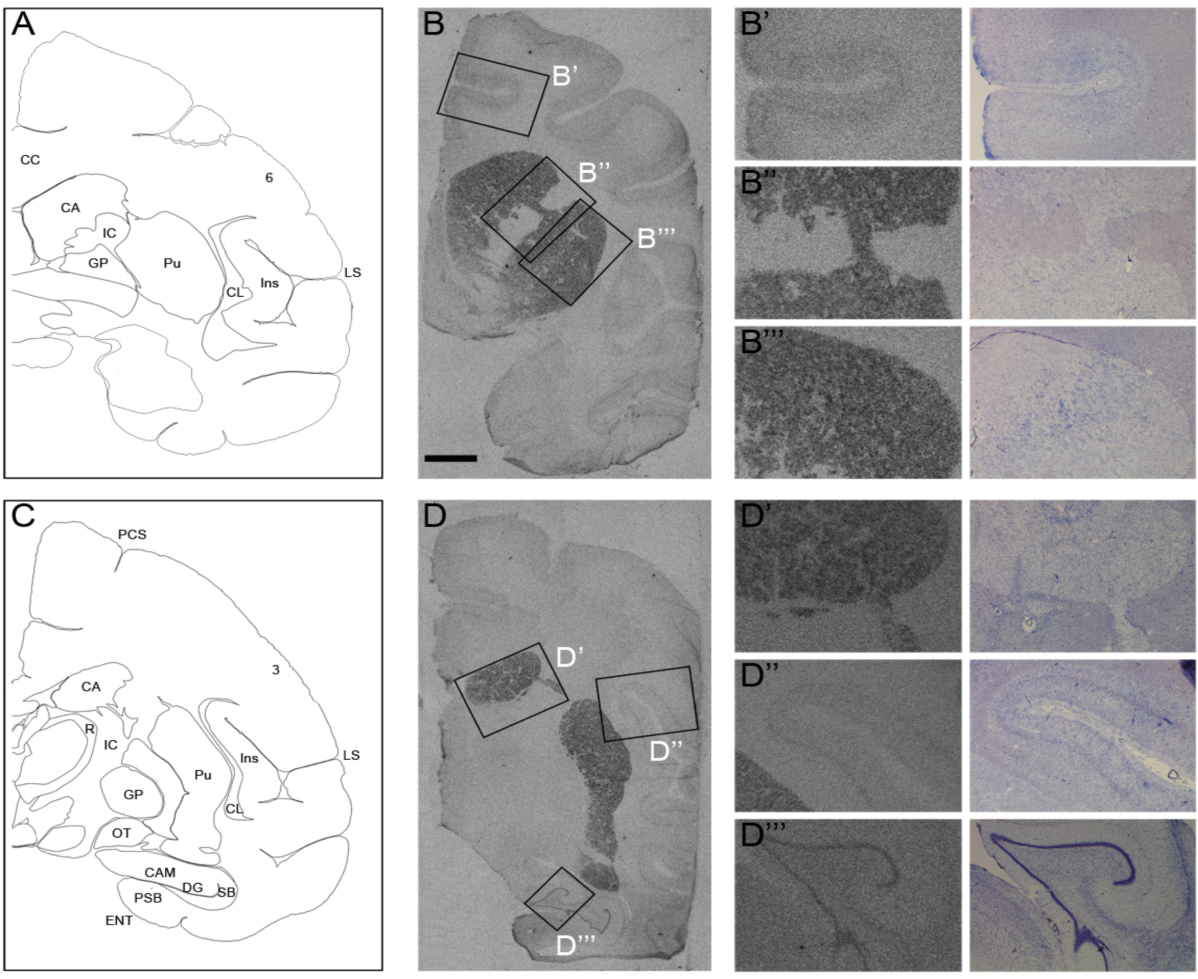
Rhes mRNA expression in Macaca mulatta adult brain. (A, C) Graphic representation of *Macaca mulatta* brain sections examined. Rostro-caudal levels are approximate and sections were obtained from www.brainmaps.org [34]. (B, D) Representative images of autoradiographic film of radioactive ISH experiments showing *Rhes* expression along the rostro-caudal extent of *Macaca mulatta adult* brain. Boxed regions in B and D are shown at higher magnification in (B’) cortical area 6 of the brain, (D’) cortical area 3 of the brain, (B”- B”‘, D’-D”) *caudate nucleus* and *putamen* of striatum, (D”‘) *cornus Ammoni* and dentate gyrus of hippocampus. Abbreviations: 3: cortical area 3; 6: cortical area 6; CA: *caudate nucleus*; CAM: *Cornus* Ammonis; CC: *corpus callosum*; CL: central lateral nucleus of the thalamus; DG: dentate gyrus; ENT: entorhinal cortex; GP: *globus pallidus*; IC: internal capsule; Ins: insula; LS: *lateral sulcus*; PCS: *precentral sulcus*; PSB: *presubiculum*; Pu: *putamen*; R: *reticular nucleus*; OT: optic tract; SB: *subiculum*. Scale bar: 5 mm (A-B, C-D); 1.75 mm (B’-B”‘, D’-D”‘).

To identify the spatial pattern of Rhes mRNA expression we performed *in situ* hybridization analysis on coronal sections of frozen brains of adult non-human primates, *Macaca Mulatta*. In agreement with previous reports in rodent and human brains [8, 9, 12], abundant levels of Rhes mRNA were observed in the striatum of macaques (Fig 2B and 2C; corresponding brain atlas tables are depicted in A and C, respectively). An enriched amount of Rhes transcript was evident along the rostro-caudal extent of the striatum in the caudate nucleus and putamen (Fig 2B”, 2B”‘, 2D’ and 2D”). In addition, consistent with previous observations in human brains [8], Rhes mRNA was also expressed in the deep layers of the cerebral cortex (Fig 2B”‘ and 2D”), *Cornu Ammonis* (CAM) and in the dentate gyrus (DG) of the *Macaca Mulatta* brain (Fig 2D”‘).

### Rhes mRNA expression levels in the brain of MPTP-treated *Macaca Mulatta*

**Fig 3:**
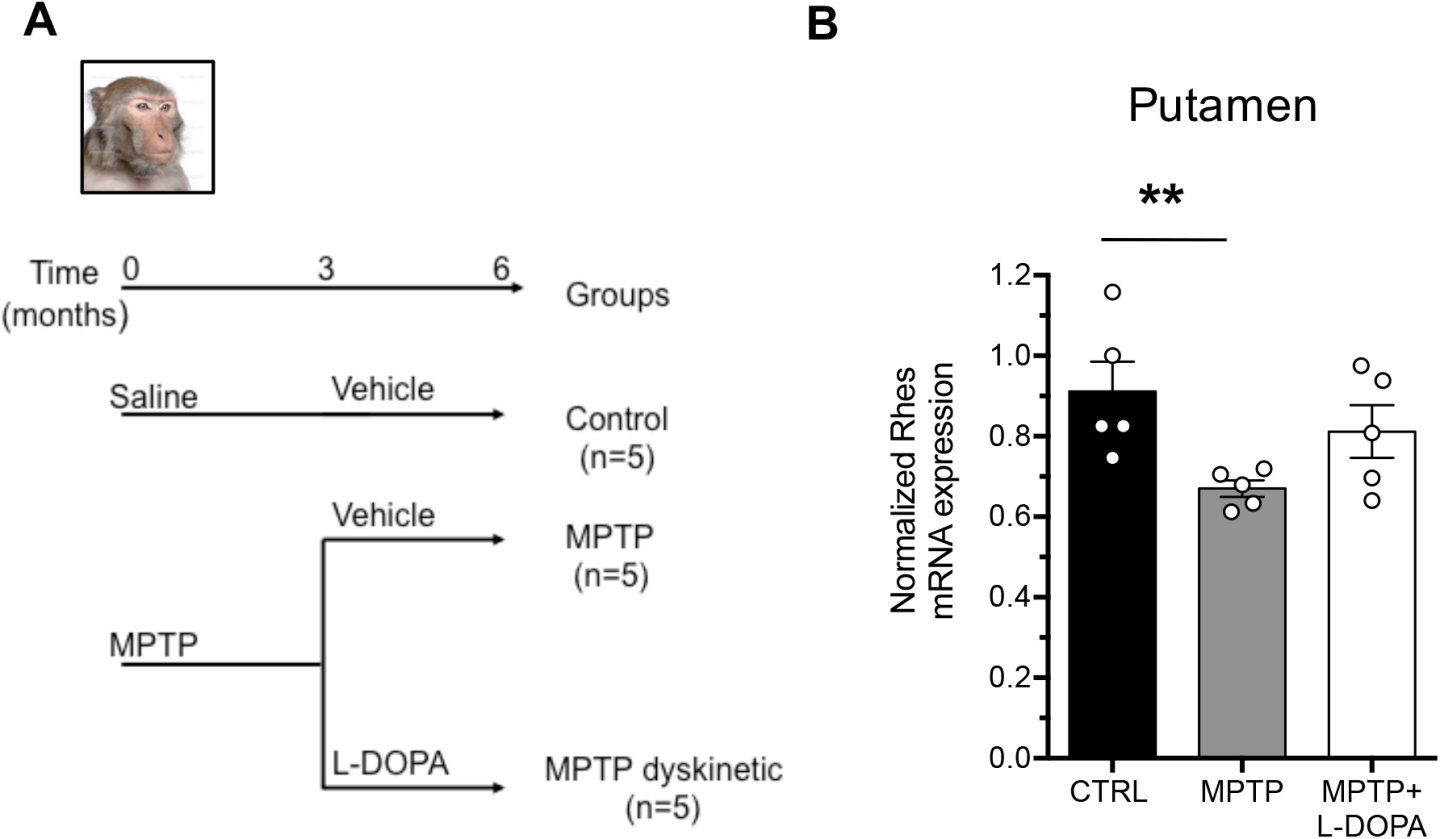
Quantitative RT-PCR analysis shows a selective reduction of Rhes mRNA levels in the MPTP macaque PD model, recovered by L-DOPA treatment. (A) Experimental design. (B) Putamen of MPTP (n = 5), MPTP+L-DOPA (n = 5) or control (n = 5) monkey groups. Data were analysed by using the Kruskal-Wallis test, followed by Mann-Whitney U test ** *p*<0.01, compared to control group. All values are expressed as mean ± SEM.

After establishing its brain localization in healthy macaques, we analysed the amount of Rhes mRNA in the putamen of monkeys treated with MPTP (Fig 3A), highly regarded as an experimental model of PD [37]. Remarkably, Kruskal-Wallis analysis displayed a significant variation of Rhes transcript levels among control vs MPTP vs MPTP+L-DOPA putamen (*P* = 0.0176; Fig 3B). In particular, Mann-Whitney test showed a significant downregulation of Rhes mRNA amount in MPTP-treated monkeys (control vs MPTP: *P* = 0.0079), that was normalized after 2-week L-DOPA supplementation (control vs MPTP+L-DOPA: *P* = 0.3095).

## Discussion

Despite an emerging interest in Rhes as a novel molecular target in striatal neuropathology, the vast majority of studies so far were performed in cell cultures and rodent models [16]. Here, we measured *Rhes* mRNA levels in a large group of *post-mortem* samples (about 200), from subjects diagnosed with SCZ, BD, PD or healthy controls, obtained from four different international brain banks. Altogether, our results indicated that Rhes mRNA levels in the putamen, hippocampus, anterior cingulate cortex, frontal and dorsolateral prefrontal cortex of SCZ patients did not significantly differ from those of healthy subjects. Similarly, in the anterior cingulate cortex of individuals suffering from BD, Rhes mRNA expression was comparable to that of non-psychiatric subjects. The lack of significant differences in Rhes mRNA expression between brain samples from controls and psychiatric patients does not rule out a possible influence of previous pharmacological medications. However, preclinical data in rats showed that chronic treatment with eticlopride, a selective D2R blocker, did not alter the striatal expression of this gene [6].

In contrast with the findings obtained in SCZ and BD brains, we found a significant reduction of Rhes transcript levels in the *post-mortem* putamen of PD patients. It’s worth mentioning that both age and PMI values are comparable between healthy controls and PD patients (NC vs PD: Age, *P* = 0.0672; PMI, *P* = 0.2494, Mann-Whitney U test; for further details, see Table 1), thus suggesting that such a downregulation is most likely associated to the pathological condition, rather than demographic sample features. On the other hand, in line with previous findings [38], dyskinetic patients analysed here showed a longer accumulated lifetime L-DOPA (*P* = 0.0408) and duration of L-DOPA treatment (*P* = 0.0391), compared to non-dyskinetic subjects (Table 1).

Consistent with PD patient observations, a similar Rhes mRNA decrease was found also in the putamen of MPTP-treated *Macaca Mulatta*. The overall downregulation of Rhes mRNA levels in the putamen of both patients and the non-human primate model is in agreement with previous findings obtained in the striatum of adult 6-OHDA-lesioned rats [6], suggesting that the regulation of Rhes gene expression by dopaminergic innervation is conserved across species. Although the biological role of this regulation is still unknown, the inhibitory influence of Rhes over the striatal dopamine-dependent responses, documented by *in vitro* and *in vivo* studies [9, 10, 15], let us hypothesize that the decrease of this G-protein may represent an event, up-stream to a complex compensatory process, able to functionally counteract the pathological reduction of dopaminergic innervation. In this view, Rhes could act as a physiological “molecular brake” for the striatal dopaminergic transmission, an idea also supported by the evidence that the lack of this GTPase triggers exaggerated motor responses in Rhes knockout mice, treated with very low doses of the dopamine releaser amphetamine [12].

In PD mouse model Rhes enhances L-DOPA-induced dyskinesia (LID), most likely through the activation of mTOR pathway [20]. Accordingly, a significant LID reduction has been observed in 6-OHDA-treated Rhes knockout mice [19, 20]. Here, despite the above preclinical observations, we did not find any significant differences in the striatal Rhes mRNA levels between dyskinetic and non-dyskinetic patients. Further studies on a larger cohort of *post-mortem* brains from PD patients with, or without, dyskinesia, are required to clarify this issue.

Notably, we report that the pattern of Rhes mRNA expression in the brain of non-human primates resembles that previously described in humans [8, 12]. Indeed, our observations in adult *Macaca Mulatta* demonstrate a prominent expression of Rhes transcript in the caudate putamen and, to a lesser extent, in the cortex, with a spatial pattern reminiscent to that in human brain [12]. Interestingly, while L-DOPA administration to MPTP-treated monkeys normalized striatal Rhes mRNA levels, this effect was not found in L-DOPA-treated PD patients. This discrepancy might be explained by a difference in dopamine levels between PD patients and MPTP-lesioned primates at the time of death or sacrifice, respectively. Indeed, while MPTP-treated macaques were killed 60 min after L-DOPA injection [39], the last dose of L-DOPA before death of PD patients was likely further in the past, and might have worn off.

However, whether or not the reported reduction of striatal Rhes mRNA expression in PD patients and MPTP-treated macaques might also reflect a decreased expression of the corresponding protein remains an issue to be addressed in future studies. In this respect, it has been shown that mRNA expression is well correlated with protein levels [40], though differentially expressed and highly expressed mRNAs correlate better with their protein product than non-differentially expressed mRNAs [41]. This is not surprising, since low expressed mRNAs and proteins have a higher degree of natural and methodological noise [42]. Rhes is highly expressed in the putamen which indicates that differences in mRNA will likely be reflected in protein levels. Furthermore, correlations of mRNA and protein in the PD putamen were previously verified for molecules such as alpha-synuclein and adenosine A2A receptors [43, 44].

A further support for a possible link between Rhes and Parkinson’s disease comes from recent findings indicating that this small GTPase is also expressed in a subset of tyrosine hydroxylase-positive neurons of the substantia nigra pars compacta, where its genetic ablation enhances vulnerability to age-dependent neuronal death in mutant mice [45]. Although the specific mechanism underpinning the relationship between Rhes and the survival of dopaminergic neurons is not clear, previous studies demonstrating its binding to beclin-1 [46] suggest a potential involvement of this G protein in cellular events implicated in neurodegeneration.

In conclusion, the present data obtained in both PD patients and experimental non-human primate model of PD suggest that the regulation of Rhes mRNA levels by dopaminergic innervation might play a functional role under physiological and pathological conditions.

## Supporting information

S1 Table Medical information about SCZ patients from HBSFRC and MRC-LNDBB brain banks. Abbreviations: HBSFRC: Human Brain Human Brain and Spinal Fluid Resource Center-Los Angeles; MRC-LNDBB: MRC London Neurodegenerative Diseases Brain Bank; SCZ: Schizophrenia.
(DOCX)

S2 Table Medical information about SCZ and BD patients from SMRI brain bank. Abbreviations: SMRI: Stanley Medical Research Institute; SCZ: Schizophrenia; BD:Bipolar Disorder.
(DOCX)
