# Supplementary material for: Decreased Rhes mRNA levels in the brain of patients with Parkinson’s disease and MPTP-treated macaques
Source: PLoS One. 2017 Jul 25;12(7):e0181677. doi: 10.1371/journal.pone.0181677 (PMC5526584; doi:10.1371/journal.pone.0181677)
Supplement: S1 Table — Abbreviations: HBSFRC: Human Brain Human Brain and Spinal Fluid Resource Center-Los Angeles; MRC-LNDBB: MRC London Neurodegenerative Diseases Brain Bank; SCZ: Schizophrenia. (DOCX) [file pone.0181677.s001.docx]

| **Sample** | **Case** | **Brain Bank** | **Diagnosis** | **Antipsychotic medication** |
| --- | --- | --- | --- | --- |
| 1 | 4448 | HBSFRC | SCZ | Quietapine |
| 2 | 4504 | HBSFRC | SCZ | Risperidone, Fluphenazine |
| 3 | 4507 | HBSFRC | SCZ | Olanzapine, Fluphenazine |
| 4 | 4649 | HBSFRC | SCZ | Unknown |
| 5 | 4723 | HBSFRC | SCZ | Risperidone, Haloperidol |
| 6 | 4730 | HBSFRC | SCZ | Unknown |
| 7 | 4804 | HBSFRC | SCZ | Thioridazine, Fluphenazine, Quietapine, Paliperidone |
| 8 | 4594 | HBSFRC | SCZ | Quietapine, Haloperidol, Risperidone |
| 9 | 4598 | HBSFRC | SCZ | Risperidone |
| 10 | 4298 | HBSFRC | SCZ | Risperidone |
| 11 | 4680 | HBSFRC | SCZ | Quietapine, Risperidone |
| 12 | 4866 | HBSFRC | SCZ | Aripiprazole, Risperidone |
| 13 | 4558 | HBSFRC | SCZ | Ziprasidone, Risperidone |
| 14 | 4610 | HBSFRC | SCZ | Risperidone |
| 15 | 4361 | HBSFRC | SCZ | Fluphenazine, Risperidone |
| 16 | 3436 | HBSFRC | SCZ | Quietapine |
| 17 | 4886 | HBSFRC | SCZ | Aripiprazole, Quietapine |
| 18 | 3739 | HBSFRC | SCZ | Quietapine |
| 19 | 3788 | HBSFRC | SCZ | Compazine, Risperidone |
| 20 | 5116 | HBSFRC | SCZ | Fluphenazine, Chlorpromazine |
| 1 | 124/94 | MRC-LNDBB | SCZ | Unknown |
| 2 | 181/94 | MRC-LNDBB | SCZ | Unknown |
| 3 | 321/94 | MRC-LNDBB | SCZ | Flupenthixol |
| 4 | 324/94 | MRC-LNDBB | SCZ | Trifluoperazine |
| 5 | 71/95 | MRC-LNDBB | SCZ | Carbamazepine |
| 6 | 128/95 | MRC-LNDBB | SCZ | Chlorpromazine |
| 7 | 152/95 | MRC-LNDBB | SCZ | Thioridazine, Fluphenazine |
| 8 | 344/95 | MRC-LNDBB | SCZ | Unknown |
| 9 | 16/93 | MRC-LNDBB | SCZ | Promazine |
| 10 | 70/93 | MRC-LNDBB | SCZ | Unknown |
| 11 | 91/93 | MRC-LNDBB | SCZ | Haloperidol |
| 12 | 186/93 | MRC-LNDBB | SCZ | Haloperidol |
| 13 | 218/93 | MRC-LNDBB | SCZ | Stelazine |
| 14 | 271/93 | MRC-LNDBB | SCZ | Chlorpromazine |
| 15 | 46/94 | MRC-LNDBB | SCZ | Trifluoperazine |

**Table S1. Medical information about SCZ patients from HBSFRC and MRC-LNDBB brain banks**

Abbreviations: HBSFRC: Human Brain Human Brain and Spinal Fluid Resource Center-Los Angeles; MRC-LNDBB: MRC London Neurodegenerative Diseases Brain Bank; SCZ: Schizophrenia.
