# Supplementary material for: Decreased Rhes mRNA levels in the brain of patients with Parkinson’s disease and MPTP-treated macaques
Source: PLoS One. 2017 Jul 25;12(7):e0181677. doi: 10.1371/journal.pone.0181677 (PMC5526584; doi:10.1371/journal.pone.0181677)
Supplement: S2 Table — Abbreviations: SMRI: Stanley Medical Research Institute; SCZ: Schizophrenia; BD:Bipolar Disorder. (DOCX) [file pone.0181677.s002.docx]

| **Sample** | **ID** | **Diagnosis** | **LifeTime antipsychotics**  **(mg of fluphenazine equivalents)** | **Sample** | **ID** | **Diagnosis** | **LifeTime antipsychotics**  **(mg of fluphenazine equivalents)** |
| --- | --- | --- | --- | --- | --- | --- | --- |
| **25** | 224 | SCZ | 100,000 | **20** | 204 | BD | 0 |
| **3** | 110 | SCZ | 180,000 | **18** | 201 | BD | 30,000 |
| **10** | 170 | SCZ | 75,000 | **23** | 217 | BD | 3,000 |
| **11** | 175 | SCZ | 130,000 | **34** | 244 | BD | 12,000 |
| **12** | 187 | SCZ | 2,500 | **69** | 373 | BD | 15,000 |
| **17** | 199 | SCZ | 15,000 | **72** | 381 | BD | 0 |
| **22** | 213 | SCZ | 20,000 | **98** | 447 | BD | 10,000 |
| **26** | 226 | SCZ | 12,000 | **1** | 39 | BD | 9,000 |
| **31** | 235 | SCZ | 350,000 | **46** | 298 | BD | 500 |
| **39** | 270 | SCZ | 20,000 | **74** | 391 | BD | 3,000 |
| **41** | 286 | SCZ | 90,000 | **102** | 456 | BD | 0 |
| **44** | 290 | SCZ | 10,000 | **5** | 113 | BD | 0 |
| **55** | 335 | SCZ | 300,000 | **8** | 155 | BD | 0 |
| **66** | 365 | SCZ | 50 | **4** | 111 | BD | 10,000 |
| **68** | 369 | SCZ | 600 | **6** | 119 | BD | 0 |
| **73** | 387 | SCZ | 120,000 | **57** | 340 | BD | 1,200 |
| **75** | 392 | SCZ | 400,000 | **32** | 450 | BD | 15,000 |
| **78** | 399 | SCZ | 50,000 | **63** | 357 | BD | 4,500 |
| **81** | 404 | SCZ | 15,000 | **14** | 192 | BD | 0 |
| **82** | 409 | SCZ | 48,000 | **61** | 354 | BD | 30,000 |
| **85** | 414 | SCZ | 15,000 | **64** | 359 | BD | 2,000 |
| **87** | 421 | SCZ | 115,000 | **76** | 393 | BD | 3,000 |
| **88** | 422 | SCZ | 70,000 | **104** | 465 | BD | 2,000 |
| **92** | 433 | SCZ | 10,000 | **2** | 42 | BD | 0 |
| **97** | 446 | SCZ | 200,000 | **21** | 212 | BD | 0 |
| **100** | 451 | SCZ | 30,000 | **58** | 342 | BD | 0 |
| **101** | 452 | SCZ | 60,000 | **62** | 356 | BD | 25,000 |
| **7** | 133 | SCZ | 35,000 | **16** | 198 | BD | 0 |
| **9** | 167 | SCZ | 20,000 | **89** | 424 | BD | 10,000 |
| **37** | 262 | SCZ | 120,000 |  |  |  |  |
| **40** | 280 | SCZ | 34,000 |  |  |  |  |
| **45** | 291 | SCZ | 200,000 |  |  |  |  |
| **47** | 299 | SCZ | 20,000 |  |  |  |  |
| **93** | 439 | SCZ | 90,000 |  |  |  |  |
| **42** | 288 | SCZ | 130,000 |  |  |  |  |
| **94** | 440 | SCZ | 18,000 |  |  |  |  |

Abbreviations: SMRI: Stanley Medical Research Institute; SCZ:Schizophrenia; BD:Bipolar Disorder.
